# A nomogramic model for predicting the left ventricular ejection fraction of STEMI patients after thrombolysis-transfer PCI

**DOI:** 10.3389/fcvm.2023.1178417

**Published:** 2023-09-08

**Authors:** Shuai Liu, Zhihui Jiang, Yuanyuan Zhang, Shuwen Pang, Yan Hou, Yipei Liu, Yuekang huang, Na Peng, Youqing Tang

**Affiliations:** ^1^ Graduate School, Guangzhou University of Chinese Medicine, Guangzhou, China; ^2^ Department of Emergency Medicine, General Hospital of Southern Theater Command, Guangzhou, China; ^3^ Department of Emergency Medicine, Guangdong Second Provincial General Hospital, Guangzhou, China; ^4^ Department of Pharmacy, General Hospital of Southern Theater Command, Guangzhou, China; ^5^ School of Pharmaceutical Sciences, Southern Medical University, Guangzhou, China; ^6^ Department of Geriatrics, General Hospital of Southern Theater Command, Guangzhou, China; ^7^ The First School of Clinical Medicine, Southern Medical University Guangzhou, Guangzhou, China

**Keywords:** STEMI, TTPCI, LVEF, prediction model, nomogram

## Abstract

**Background:**

The prognosis of ST-segment elevation myocardial infarction (STEMI) is closely linked to left ventricular ejection fraction (LVEF). In contrast to primary percutaneous coronary intervention (PPCI), thrombolysis-transfer PCI (TTPCI) is influenced by multiple factors that lead to heterogeneity in cardiac function and prognosis. The aim of this study is to develop a nomogram model for predicting early LVEF in STEMI patients with TTPCI, based on routine indicators at admission.

**Method:**

We retrospectively reviewed data from patients diagnosed with STEMI at five network hospitals of our PCI center who performed TTPCI as door-to-balloon time (the interval between arrival at the hospital and intracoronary balloon inflation) over 120 min, from February 2018 to April 2022. Categorical variables were analyzed using Pearson *χ*^2^ tests or Fisher exact tests, while Student's *t*-test or Mann–Whitney *U*-test was used to compare continuous variables. Subsequently, independent risk factors associated with reduced LVEF one week after TTPCI were identified through comprehensive analysis by combining All-Subsets Regression with Logistic Regression. Based on these indicators, a nomogram model was developed, and validated using the area under the receiver operating characteristic (ROC) curve and the Bootstrap method.

**Results:**

A total of 288 patients were analyzed, including 60 with LVEF < 50% and 228 with LVEF ≥ 50%. The nomogram model based on six independent risk factors including age, heart rate (HR), hypertension, smoking history, Alanine aminotransferase (ALT), and Killip class, demonstrated excellent discrimination with an AUC of 0.84 (95% CI: 0.78–0.89), predicted C-index of 0.84 and curve fit of 0.713.

**Conclusions:**

The nomogram model incorporating age, HR, hypertension, smoking history, ALT and Killip class could accurately predict the early LVEF ≥ 50% probability of STEMI patients undergoing TTPCI, and enable clinicians' early evaluation of cardiac function in STEMI patients with TTPCI and early optimization of treatment.

## Background

STEMI represents the most serious manifestation of coronary atherosclerosis disease, with an in-hospital heart failure rate of 14.2% and a mortality rate ranging from 3% to 4% (1). Current reperfusion modalities for STEMI patients include thrombolysis, PPCI or coronary artery bypass grafting (CABG) (2). However, TTPCI is recommend by guidelines as an effective reperfusion strategy for patients with expected door-to-balloon time over 120 min at non-PCI center (3, 4). Nevertheless, multiple intermediate steps involving thrombolysis, transfer with ambulance, evaluation before PCI and the post-thrombolytic complications occurrence such as thrombolysis failure, reperfusion arrhythmias, and reinfarction post thrombolysis, as well as differences in emergency medical care contribute to the heterogeneity and uncertainty of prognosis in these populations (5). Several studies have shown a strong correlation of LVEF reduction after PCI with in-hospital mortality as well as major adverse cardiovascular events (MACEs) for STEMI patients (6–8). Additionally some biomarkers such as mid-regional precursor atrial natriuretic peptide (MR-proANP), brain natriuretic peptide (BNP) have reported the predictive value for LVEF of STEMI patients post-PCI (9–11); but few predictive model has been established for STEMI patients' LVEF after TTPCI.

This study aims to develop a nomogram model predicting early LVEF for patients with TTPCI, according to routine clinical and laboratory indicators at the time of entering our PCI center, which can assist clinical physician in early evaluation of cardiac function, guide the timing of PCI treatment, optimize protection strategies and improve the prognosis.

## Methods

### Patients

We conducted a retrospective analysis of data from patients with STEMI who underwent thrombolysis followed by at the regional coordination chest pain center in the General Hospital of Southern Theater Command of PLA between February 2018 and April 2022. All included patients were diagnosed STEMI at five network hospitals of our center with door-to-balloon time of over 120 min. Patients with incomplete clinical data, or first medical contact time (FMC) >12 h, or pre-existing cardiac insufficiency were excluded from the analysis. All patients were given 300 mg aspirin and 300–600 mg clopidogrel immediately after diagnosis, followed by thrombolysis with teneprase (Guangzhou Mingkang Biological Co., Ltd., State Drug Administration S20150001, 16 mg, 10 s intravenous), and anticoagulation with normal heparin sodium or enoxaparin. Vital signs of the patients were observed for 30–120 min after thrombolysis, and then transferred to the General Hospital of Southern Theater Command of PLA. If thrombolysis failed, PCI was performed immediately. Otherwise, coronary angiography or PCI was carried out within 24 h. M-mode echocardiographic measurement was conducted one week after TTPCI and patients were categorized into two groups according to LVEF: reduced group (LVEF < 50%) and preserved group (LVEF ≥ 50%).The formula used for calculation was as follows: LVEF = (left ventricular end-diastolic volume - left ventricular end-systolic volume)/L left ventricular end-diastolic volume × 100%.This study has been approved by the Medical Ethics Committee of the General Hospital of Southern Theater Command of PLA (No. NZLLKZ2022035).

### Data collection and statistical analysis

A total of 57 variables were included: (1) demographic data [age, gender, weight, heart rate (HR), etc.]; (2) epidemiological data [hypertension, diabetes, coronary artery disease (CAD), smoking history, alcohol intake, etc.]; (3) time efficiency metrics (first medical contact (FMC), door-to-needle (D-to-N), door-to-balloon (D-to-B), etc.); (4) angiographic features (number and distribution of offender vessels, TIMI blood flow grading, etc.); and (5) clinical and laboratory data. The information was obtained from the database of our chest pain center as well as the electronic medical record system and interventional procedure management system.

Categorical variables were presented as frequency percentages while continuous variables were reported either as median with min-max or mean ± standard deviation. Prior to statistical analysis for normality assessment of continuous variables we used Shapiro–Wilk test. For categorical variables, Pearson's *χ*^2^ test or Fisher's exact test was employed while continuous variables were employed either by Student's *t*-test or Mann–Whitney *U*-test as appropriate.

All variables were screened by Backward Stepwise Regression according to the Akaike's Information Criterion (AIC). When the AIC value stopped declining, the full subset regression was used to screen for the variable with the largest adjusted R-squared. The odds ratio (OR) and its corresponding 95% confidence interval (CI) were calculated by univariate logistic regression and multivariate logistic regression respectively. The nomogram model was developed with those independent factors. The prediction accuracy was internally verified using the bootstrap self-sampling method, and the results were visually presented in a calibration curve. The discrimination ability of the nomogram was assessed by the receiver operating characteristic (ROC) curve, and a calibration plot was used to determine the degree of agreement between predicted and observed outcomes. The goodness of fit was evaluated using the Hosmer-Lemeshow test.Finally, the decision curve analysis (DCA) was used to assess the clinical utility of the nomogram. All tests were two-sided with an alpha level of 0.05. All data management and statistical analyses were performed using IBM SPSS V24: IBM Corp. Released 2016. IBM SPSS Statistics for Windows, Version 24.0. Armonk, NY: IBM Corp. (You can check the details via https://www.ibm.com/support/pages/howcite-ibm-spss-statistics-or-earlier-versions-spss) and the R software, version 4.1.2.

## Results

### Characteristics of STEMI patients

**Figure 1:**
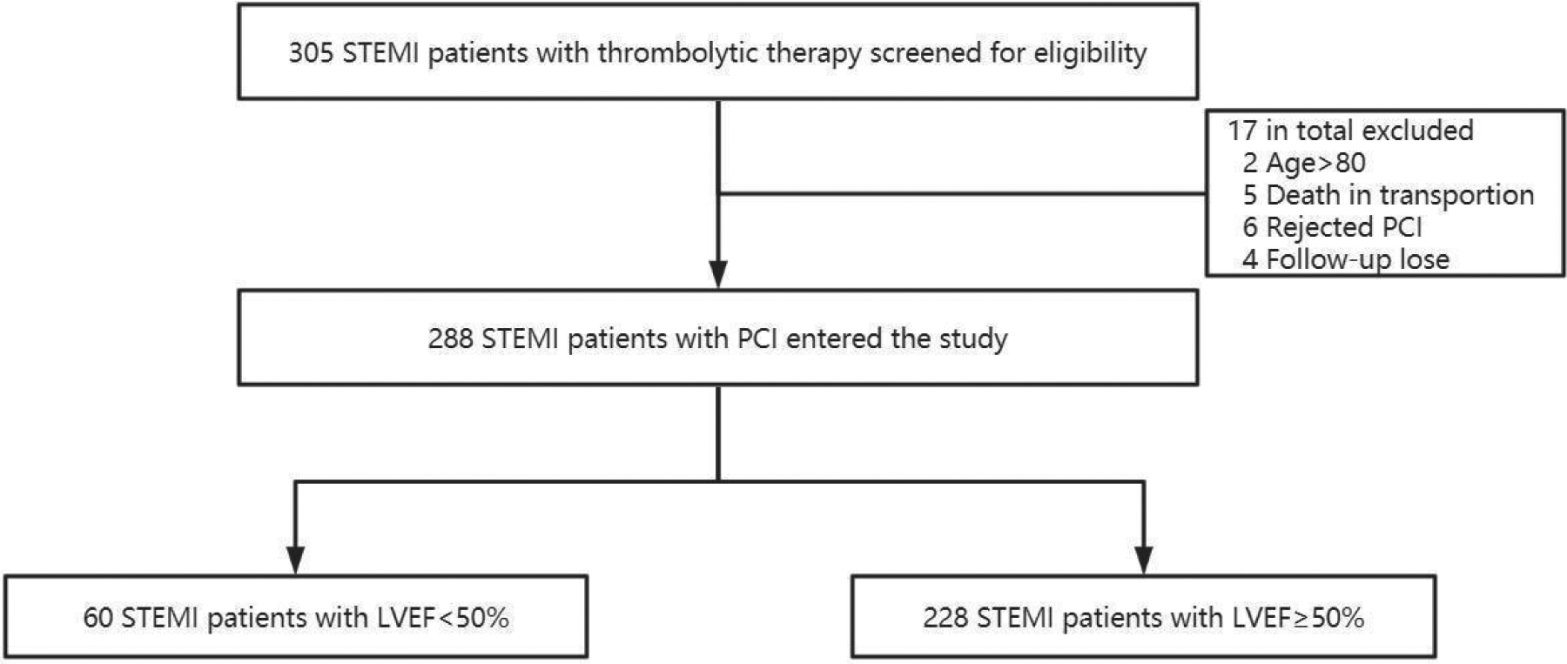
Flow chart of the study population selection. A total of 305 patients diagnosed with STEMI in primary hospitals and treated with pharmacological thrombolysis and reperfusion strategy were included in this study. However, 17 patients were excluded because of age >80 years, death in transportion, rejected PCI, or missing data, leaving 288 patients who were divided into two groups according to LVEF measured by echocardiography within 1 week after TTPCI.

From 1 February 2018 to 30 April 2022, our center received a total of 305 STEMI patients who had undergone thrombolysis in network hospitals prior to transfer for PCI. Seventeen cases were excluded due to age (<18 years or >80 years), death during transfer, patient refusal of PCI procedure, and incomplete data, and the study ultimately enrolled a total of 288 patients (Figure 1). Among the selected subjects, the LVEF reduction group included 54 (90%) male and 6 (10%) female, while the LVEF preservation group consisted of 212 (93%) males and 16 (7%) females.

There were significant differences in HR, smoking history, thrombolytic recanalization, chest pain relief, reperfusion arrhythmia, congestive heart failure, Killip class, CCU time, length of stay, thirty-day cerebral infarction, thirty-day mortality at admission between the LVEF-reduced (LVEF < 50%) group and LVEF-preserved (LVEF ≥ 50%) group (*p* < 0.05) (Table 1).

**Table 1:** Clinical characteristics of patients in the LVEF-reduced and LVEF-preserved groups at admission.

Variable	LVEF reduction (*n* = 60)	LVEF preservation (*n* = 228)	*p*
Baseline characteristics			
Age, years	57.4 ± 12.1	54.4 ± 10.8	.058
Sex, Male (%)	54 (90%)	212 (93%)	.617
HR, rate/min	88.0 (80.0, 98.5)	78.0 (68.0, 87.5)	<.001*
SBP, mmHg	124.9 ± 21.9	129.0 ± 21.4	.191
Comorbidities			
Hypertension, *n* (%)	18 (30%)	98 (43%)	.094
Diabetes, *n* (%)	15 (25%)	48 (21.1%)	.629
CVD, *n* (%)	14 (23.3%)	58 (25.4%)	.867
Smoking history, *n* (%)	38 (63.3%)	182 (79.8%)	.012*
Alcohol intake, *n* (%)	20 (33.3%)	73 (32%)	.969
Anticoagulant therapy			
UFH, *n* (%)	27 (45%)	84 (36.8%)	.314
Enoxaparin, *n* (%)	33 (55%)	144 (63.2%)	.686
Symptoms			
Thrombolytic recanalization, *n* (%)	36 (60%)	189 (82.9%)	<.001*
Chest pain relief, *n* (%)	35 (58.3%)	182 (79.8%)	.001*
Reperfusion arrhythmia, *n* (%)	15 (25%)	26 (11.4%)	.013*
>50%ST-segment resolution, *n* (%)	43 (71.7%)	186 (81.6%)	.130
Bleeding, *n* (%)	1 (1.7%)	5 (2.2%)	1.000
Congestive heart failure, *n* (%)	19 (31.7%)	19 (8.3%)	<.001*
Shock, *n* (%)	5 (8.3%)	5 (2.2%)	.055
Killip class			
Killip class 1, *n* (%)	39 (65%)	209 (91.7%)	<.001*
Killip class 2, *n* (%)	13 (21.7%)	17 (7.5%)	
Killip class 3, *n* (%)	1 (1.7%)	0 (0%)	
Killip class 4, *n* (%)	7 (11.7%)	2 (0.9%)	
PCI Culprit vessel			
Numbers	1.0 (1.0, 3.0)	1.0 (1.0, 2.5)	.304
LAD, *n* (%)	51 (85%)	164 (71.9%)	.057
LCA, *n* (%)	23 (38.3%)	72 (31.6%)	.403
RCA, *n* (%)	32 (53.3%)	126 (55.3%)	.903
LMC, *n* (%)	3 (5%)	6 (2.6%)	.602
Microangiopathy, *n* (%)	38 (63.3%)	123 (53.9%)	.247
TIMI-flow before PCI			
TIMI-flow 0, *n* (%)	7 (11.7%)	22 (9.6%)	.775
TIMI-flow 1, *n* (%)	4 (6.7%)	9 (3.9%)	
TIMI-flow 2, *n* (%)	6 (10%)	23 (10.1%)	
TIMI-flow 3, *n* (%)	43 (71.7%)	174 (76.3%)	
Outcome indicator			
CCU, days	4.0 (3.0, 4.0)	3.0 (2.0, 4.0)	<.001*
Length of stay, days	7.5 (6.0,10.0)	6.0 (5.0, 8.0)	<.001*
30-day cerebral infarction, *n* (%)	3 (5%)	0 (0%)	.007*
30-day R-AMI, *n* (%)	2 (3.3%)	13 (5.7%)	.683
30-day mortality, *n* (%)	3 (5%)	0 (0%)	.007*
Time intervals			
FMC, min	85.0 (53.0, 192.0)	71.5 (45.0, 152.5)	.259
D-to-N, min	27.0 (23.0, 37.0)	28.0 (22.0, 39.0)	.952
Departure time, min	86.0 (56.0, 122.0)	90.0 (59.0, 136.0)	.532
D-to-B, min	357.0 (226.0,1,150.5)	643.0 (236.5, 1,187.5)	.270

The data are expressed in median (min-max) or number (%),when appropriate.

HR, heart rate; SBP, systolic blood pressure; CVD, cardiovascular disease; UFH, unfractionated heparin; TIMI, thrombolysis in myocardial infarction; LAD, left anterior descending; LCA, left circumflex artery; RCA, right coronary artery; LMC, left main coronary; CCU, cardiology care unit; R-AMI, recurrent acute myocardial infarction; FMC, first medical contact; D-to-N, door to needle; D-to-B, door to balloon.

*p-*values were estimated by *χ*^2^ test, Fisher's exact test, or McNemar test for categorical variables and Mann–Whitney *U* test for continuous variables as appropriate.

* *p* < 0.05 was considered statistically significant.

Additionally, the two groups showed great distinctions in white blood cell count (WBC), neutrophil count, neutrophil-to-lymphocyte ratio (NLR), alanine aminotransferase (ALT), blood urea nitrogen (BUN), triglyceride (TG), and D-dimer within 24 h after admission (Table 2).

**Table 2:** Laboratory parameters of patients in the LVEF-reduced and LVEF-preserved groups at admission.

Variable	LVEF reduction (*n* = 60)	LVEF preservation (*n* = 228)	*p*
WBC, ×10^9^/L	13.1 (10.8, 16.5)	11.6 (9.3, 14.6)	.004*
Neutrophil, ×10^9^/L	11.2 (8.5, 14.5)	9.4 (7.2, 12.1)	.004*
Lymphocyte, ×10^9^/L	1.4 (0.9, 2.0)	1.5 (1.1, 1.9)	.479
NLR	8.5 (5.1, 11.7)	6.6 (4.2, 9.7)	.029*
Hb, g/L	145.0 (131.5, 158.5)	144.0 (133.0, 151.0)	.231
PLT, ×10^9^/L	240.5 (190.5, 272.0)	232.0 (198.5, 269.0)	.699
TBil, µmol/L	9.4 (7.4, 12.1)	8.9 (6.4, 11.5)	.189
DBil, µmol/L	3.8 (3.1, 5.6)	3.5 (2.8, 4.5)	.076
Alb, g/L	41.0 (38.8, 43.3)	41.2 (38.6, 43.6)	.911
ALT, U/L	48.0 (31.0, 88.5)	32.0 (21.0, 50.0)	<.001*
BUN, mmol/L	5.0 (4.1, 6.6)	4.7 (3.8, 5.5)	.017*
Cr, µmol/L	77.5 (69.0, 93.5)	79.5 (70.0, 92.0)	.747
Glu, mmol/L	6.3 (5.2, 8.4)	5.8 (5.1, 7.1)	.232
TC, mmol/L	5.0 (3.3, 5.9)	4.4 (3.6, 5.2)	.114
TG, mmol/L	1.7 (1.0, 2.4)	1.4 (1.0, 2.0)	.046*
HDL, mmol/L	1.2 (0.9, 1.5)	1.1 (0.9, 1.3)	.233
LDL, mmol/L	3.8 (2.4, 4.8)	3.2 (2.5, 4.3)	.185
PT, s	13.2 (12.8, 14.3)	13.3 (12.9, 13.9)	.622
INR	1.0 (1.0, 1.1)	1.0 (1.0, 1.1)	.782
APTT, s	51.1 (38.9, 83.2)	47.9 (41.2, 78.4)	.948
TT, s	32.3 (19.8, 231.3)	37.2 (20.4, 240.0)	.713
Fib, g/L	3.2 (2.7, 3.7)	3.2 (2.7, 3.8)	.727
D.Dimer, µg/L	1,668.5 (798.0, 3,550.0)	983.5 (531.0, 1,895.0)	.002*
BNP, pg/ml	122.1 (64.2, 296.2)	82.3 (48.0, 237.1)	.056
MYO, µg/L	400.0 (209.5, 500.0)	366.1 (84.0, 500.0)	.092
CK.MB, µg/L	80.0 (11.7, 100.0)	48.6 (9.7, 80.0)	.113
cTnI, µg/L	21.1 (0.5, 50.0)	4.6 (0.3, 36.7)	.056

The datas are expressed in median (min-max) or number (%), when appropriate.

WBC, white blood cell; NLR, neutrophil-to-lymphocyte ratio; Hb, hemoglobin; PLT, platelet; TBil, total bilirubin; DBil, direct bilirubin; Alb, albumin; ALT, alanine aminotransferase; BUN, blood urea nitrogen; Cr, creatinine; Glu, glucose; TC, total cholesterol;TG, triglyceride; HDL, high-density lipoprotein; LDL, low-density lipoprotein; PT, prothrombin time; INR, international normalized ratio; APTT, activated partial thromboplastin time; TT, thrombin time; BNP, brain natriuretic peptide; MYO, myohemoglobin; CK-MB, creatine kinase isoenzyme; cTnI, cardiac troponin.

*p-*values were estimated by *χ*^2^ test, Fisher's exact test, or McNemar test for categorical variables and Mann–Whitney *U* test for continuous variables as appropriate.

* *p* < 0.05 was considered statistically significant.

### Nomogram construction

**Figure 2:**
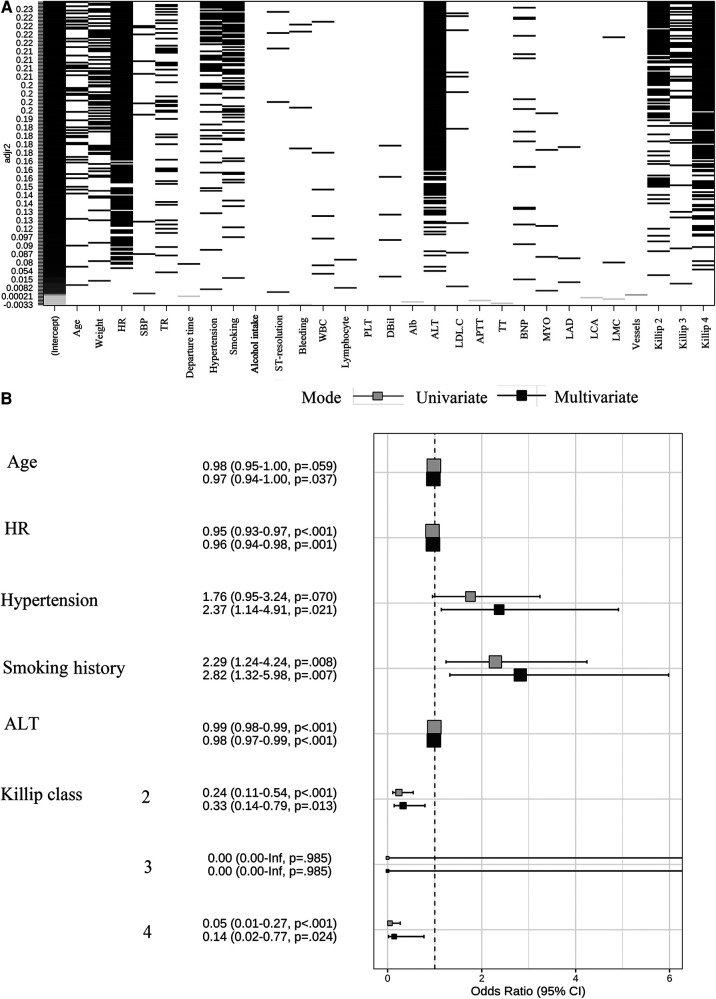
(**A**) Screening plot of 26 variables by all-subsets regression, HR, heart rate; SBP, systolic blood pressure; TR, thrombolytic recanalization; WBC, white blood cell; PLT, platelet; dBil, direct bilirubin; Alb, albumin; ALT, alanine aminotransferase; LDL.C, low-density lipoprotein cholesterin; APTT, activated partial thromboplastin time; TT, thrombin time; BNP, brain natriuretic peptide; MYO, myohemoglobin; LAD, left anterior descending; LCA, left circumflex artery; LMC, left main coronary. (**B**) Regression coefficient of independent risk factors and forest plots. A total of six independent variables were obtained through both univariate and multivariate logistic regression. OR, odds ratios; 95% CI, 95% confidence interval.

**Figure 3:**
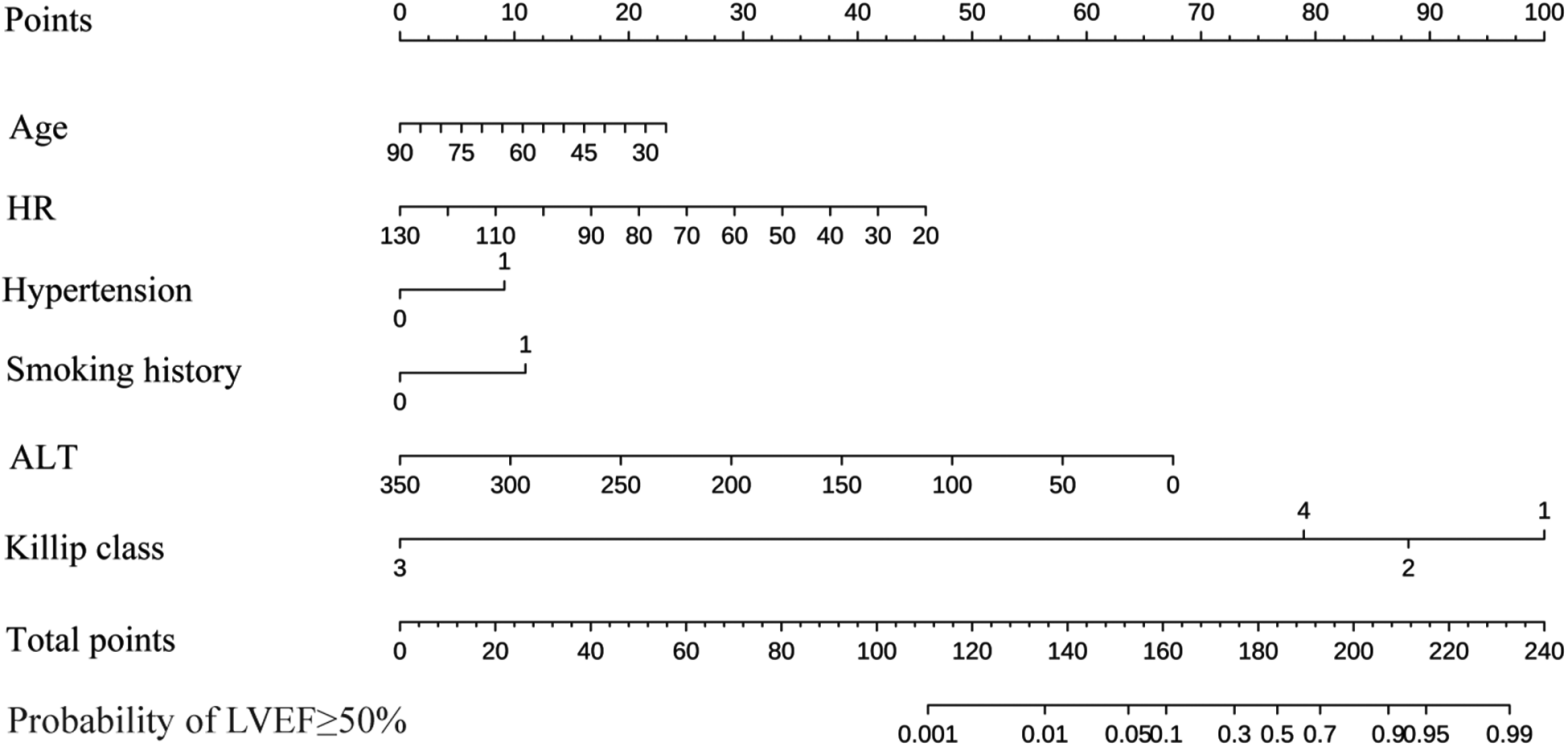
A nomogram for predicting the LVEF ≥ 50% probability of STEMI patients with PCI. Age, years; HR, heart rate, rate/min; Hypertension; Smoking history; ALT, alanine aminotransferase, U/L; Killip class. Instructions for using the nomogram: (1) Draw a vertical line based on the value of each variable to obtain the corresponding point; (2) Add all six points to obtain the total point; (3) Draw a vertical line based on the total point to determine the estimated the LVEF ≥ 50% probability one week after TTPCI.

By the full subset regression analyses, we got a total of 26 potential risk factors (Figure 2A). Six variables were identified as independent risk factors, including age (OR: 0.97; 95% CI: 0.94–1.00; *p *= 0.037); HR (OR: 0.96; 95% CI: 0.94–0.98; *p *= 0.001); hypertension (OR: 2.37; 95% CI: 1.14–4.91; *p *= 0.021); smoking history (OR: 2.82; 95% CI: 1.32–5.98; *p *= 0.007); ALT (OR: 0.98; 95% CI: 0.97–0.99; *p *< 0.001); and Killip class [Killip class 2 (OR: 0.33; 95% CI: 0.14–0.79; *p *= 0.013), Killip class 3 (OR: 0; 95% CI: 0.00-Inf; *p *= 0.985), Killip class 4 (OR: 0.14; 95% CI:0.02–0.77; *p *= 0.024)]. They were utilized to build the nomogram for prediction of LVEF ≥ 50% probability in STEMI patients with TTPCI (Figure 2B). The nomogram based on the six independent risk factors is shown in Figure 3. The “Total points” in the model represents the probability of predicting LVEF ≥ 50% for the STEMI patients in the first week after TTPCI.

### Evaluation of the nomogram

**Figure 4:**
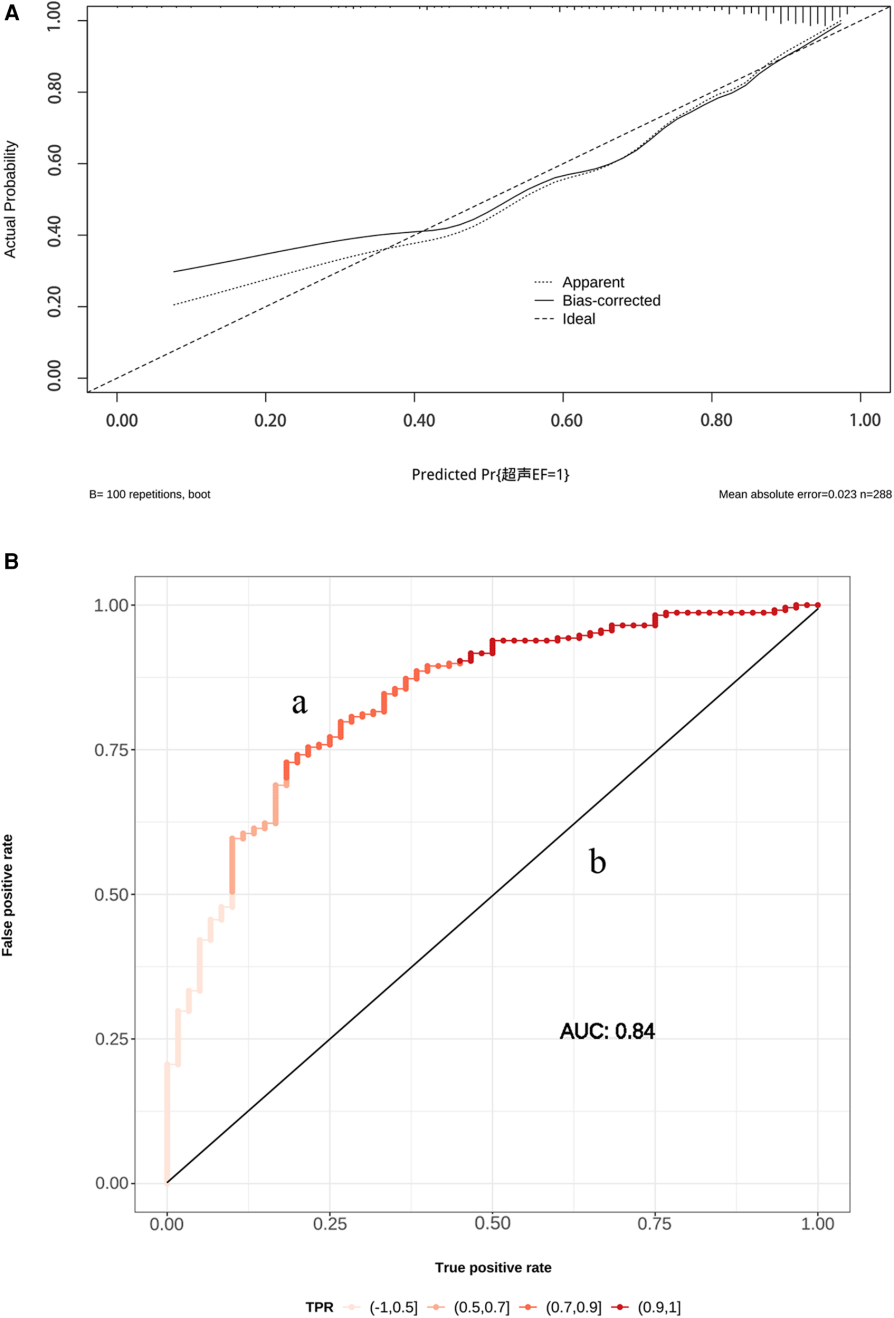
(**A**) The receiver operating characteristic (ROC) curve (a) and calibration curve (b) for the established nomogram. OR, odds ratios; AUC, area under the curve; 95% CI, 95% confidence interval. Calibration curve reflects the extent to which the model correctly estimates the absolute probability or agreement between the predicted probability and observed outcomes. (**B**) The y-axis represents the actual LVEF ≥ 50% probability. The x-axis represents the predicted LVEF ≥ 50% probability. The black dot at the top represents the prediction probability corresponding to the actual observation, the black dotted line represents the ideal predicted value, and the solid line represents the actual predicted value.

**Figure 5:**
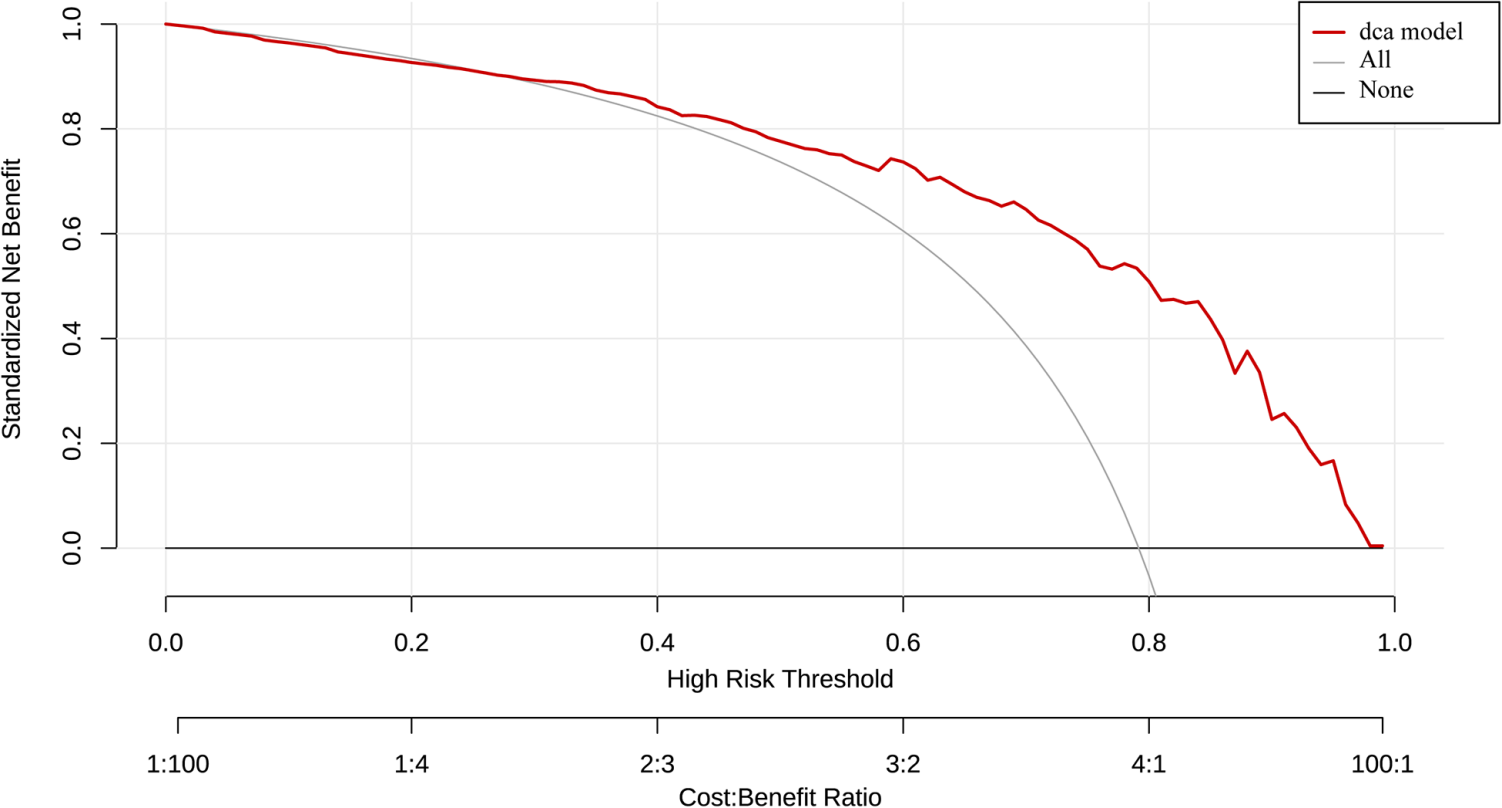
Determination of decision point via decision curve analysis (DCA) and clinical impact curve. (1) Decision curve for the prediction model. The decision curve analysis graphically shows the clinical usefulness of the nomogram based on a continuum of potential thresholds for LVEF ≥ 50% probability falling (x-axis) and the net benefit of using the nomogram to stratify patients (y-axis). Net benefit curves are plotted across probability thresholds for 6 options: “all” assume all patients have LVEF < 50%, “none” assume no patients have LVEF < 50%. Net benefit = (true positives/N)-(false positives/N) × (weighting factor). Weighting factor = threshold probability/(1-threshold probability). (2) Clinical impact curve for nomogram. The red line shows the total number who would be deemed as high risk of LVEF < 50% for each risk thresh.

The AUC of ROC curve for the nomogram was 0.84 (95% CI: 0.78–0.89) (Figure 4A). According to the Hosmer-Lemeshow goodness-of-fit test, the nomogram was demonstrated a satisfactory fit (*p *= 0.713), with a Mean Absolute Error (MAE) of 0.023 for the calibration curve (Figure 4B), which indicated that the prediction model exhibits strong predictive capabilities. Additionaly, the decision curve analysis (DCA) demonstrated that the net benefit of the prediction model was significantly higher compared with the two extreme conditions (Figure 5), suggesting its superior overall net benefit in predicting LVEF among patients with STEMI.

## Discussion

Studies have indicated that LVEF of STEMI patient is closely associated with in-hospital and long-term mortality as well as the occurrence of MACE (6–8). Multiple factors can influence early LVEF in patients with STEMI. Arso et al. demonstrated that TTPCI had a significantly higher incidence of in-hospital cardiovascular events compared with PPCI due to variability in patient baseline status, thrombolysis and execution of the transfer process, ultimately leading to greater variability in cardiac function (12). Currently, a multitude of studies (13–15) have utilized single laboratory indicators or demographic data to prognosticate cardiac function in STEMI patients following PCI. However, despite its simplicity and convenience, the overall predictive performance remains suboptimal due to various influencing factors. Our study reported a nomogram for early prediction of LVEF ≥ 50% probability following TTPCI in STEMI patients, incorporating three baseline parameters (age, smoking history, and hypertension) as well as three clinically available measures (HR, ALT, and Killip class).These multidimensional measures collectively provided a comprehensive estimation of the probability of LVEF ≥ 50%. The research error had been eliminated, leading to significant enhancement in the predictive performance of the model.

Age was an independent risk factor for reduced LVEF in STEMI patients after TTPCI in our study. Advanced age leads to impaired endothelial cell function and diffuse coronary artery calcification, increasing the risk of coronary microvascular obstruction (13), which is considered an independent risk factor for unfavorable consequences including frequent coronary events, left ventricular hypertrophy and heart failure in acute myocardial infarction (AMI) (16, 17).Qin et al. found that age ≥75 years was an independent risk factor for heart failure readmission within 30 days in patients treated with PCI, which is similar to our results (18). Therefore, a more aggressive strategy of hemodynamic reconstruction and cardioprotection in elderly STEMI patients is crucial for protection of cardiac function and long-term prognosis.

Smoking history is the well-established independent risk factors which accounts for almost 50% of STEMI patients (19), but its impact on STEMI prognosis remains uncertain. Some studies illustrate that smokers are more sensitive to thrombolytic therapy and experience lower mortality rates in comparison to non-smokers. This is probably related to the fact that the component of coronary obstruction in smokers is mainly acute thrombus rather than chronic atherosclerotic plaque, known as “the smoker's paradox” (20). However, other studies suggest that smoking causes a 2-fold increase in both AMI morbidity and mortality (21, 22). Elderly AMI patients who smoke are particularly vulnerable to long-term mortality (23). In our study, we did not found that smoking had an effect on the reduction of LVEF after TTPCI, which does not negate the damaging outcome that smoking on the cardiovascular system. Prospective studies with larger sample sizes are needed to further observe the relationship between smoking and outcome after TTPCI.

Hypertension is another important factor in AMI complicated by left ventricular dysfunction or congestive heart failure (24, 25). The related mechanisms include: (1) Elevated shear stress in blood leads to impaired vascular endothelial function, thereby compromising the ability of endothelium-mediated hyperpolarization to regulate contraction of smooth muscle cells (26); (2) increased nitric oxide production and inflammatory factors released from vascular endothelial cells in hypertensive patients lead to coronary microcirculatory dysfunction (27); and (3) oxidative stress caused by excessive bioavailability of reactive oxygen species (ROS) in hypertensive patients aggravates vascular damage (28). Thus, hypertension may result in more severe myocardial ischemia and increased cardiac load, thereby affecting cardiac function in STEMI patients.However, our results did not show that hypertension caused a reduction in LVEF for the pupulation with TTPCI. This maybe explained by the cause of a compensatory increase in myocardial systolic function (29). Moreover, standard manage of TTPCI significantly alleviate the effect of ischemia on cardiac function.

Interestingly, our study has shown that elevated ALT is an independent risk factor for predicting a decline in LVEF among STEMI patients. This is because liver receives approximately 25% of the cardiac output (CO) and is more sensitive to hypoperfusion resulting from reduced CO or hepatic artery blood flow (30). Furthermore, decreased CO often leads to hepatic venous stasis, which is the pathological basis of acute cardiogenic liver injury (31). Therefore, ALT reflects the state of organ perfusion and congestion based on cardiac pump function. A clinical trial (32) involving 105 patients with reduced ejection fraction found that AST/ALT ratio independently predicted the severity of cardiac dysfunction while a large study (33) also found that elevated ALT not only strongly correlated with MACEs, but also served as an important predictor of long-term mortality in AMI patients. Therefore, the inclusion of ALT as a predictor in the model reflects the organ-organ interactions in STEMI patients with early cardiac decompensation.

Our study demonstrates that an increased HR is hugely associated with the occurrence of reduced LVEF after TTPCI. HR is a predictor of heart failure and MACEs for AMI patients (34–36).The main pathophysiological mechanisms of HR abnormalities in STEMI patients are related to sympathetic excitation and vagal inhibition (37, 38). The equilibrium of HR is pivotal in upholding the electrical stability of ventricular myocardium and averting the emergence of lethal ventricular arrhythmias (39). A previous study confirmed that heart rate variability (HRV) (40) and heart rate kinetics after AMI are enormously correlated with survival in patients with lower LVEF (41). The Killip class is a widely used to evaluate severity of heart failure after AMI (42). Numerous studies have confirmed the close relationship between higher Killip class and reduced LVEF in STEMI patients (43–45), suggesting higher Killip class increases risk of death (46). In addition, Killip class predicts impaired left ventricular systolic function in AMI patients (43). Our study found that the higher the Killip class was associated with lower the LVEF in STEMI patients, consistent with the previous findings (47), showing the Killip class (OR: 1.449, 95% CI: 1.090–1.928, *p *= 0.011) as an independent predictor of reduced LVEF (≤45%) at hospital discharge.

In this study, we collected real clinical and laboratory data from STEMI patients who underwent regional cooperative TTPC. We meticulously analyzed the data to eliminate confounding factors, including competing risks. Furthermore, we observed that a combination of old age, elevated heart rate, increased ALT levels, and high Killip class can be suggested a lower probability of LVEF ≥ 50% one week after TTPCI. However, the study had several limitations as well. Firstly, it should be noted that it was a retrospective study and limited by a relatively small sample size. Secondly, the nomogram has not undergone external validation in another cohort of STEMI patients, and its predictive performance requires further confirmation. Thirdly, the LVEF is a relatively single endpoint indicator; thus expanding the sample size is necessary to explore predictive model for long-term prognosis of STEMI patients.

## Conclusions

In the study, we developed a nomogram that could accurately predicts the risk of early LVEF in STEMI patients with TTPCI, using easily accessible clinical indicators such as age, HR, hypertension, smoking history, ALT and Killip class. Clinicians can focus on early risk stratification and cardiac function protection with the nomogram so as to select appropriate reperfusion strategies in a timely manner. More prospective studies are expected to further confirm the early benefits of the model for a larger population.

## Data Availability

The original contributions presented in the study are included in the article/Supplementary Material, further inquiries can be directed to the corresponding authors.
